# CTAB Enhanced Room-Temperature Detection of NO_2_ Based on MoS_2_-Reduced Graphene Oxide Nanohybrid

**DOI:** 10.3390/nano12081300

**Published:** 2022-04-11

**Authors:** Wenbo Li, Hao Li, Rong Qian, Shangjun Zhuo, Pengfei Ju, Qiao Chen

**Affiliations:** 1 National Center for Inorganic Mass Spectrometry in Shanghai, Shanghai Institute of Ceramics, Chinese Academy of Sciences, Shanghai 200050, China; hymnek@hotmail.com (W.L.); lh960714211@163.com (H.L.); sjzhuo@mail.sic.ac.cn (S.Z.); 2 School of Material Science and Engineering, University of Shanghai for Science and Technology, Shanghai 200093, China; 3 Center of Materials Science and Optoelectronics Engineering, University of Chinese Academy of Sciences, Beijing 100049, China; 4 Shanghai Aerospace Equipment Manufacturer, Shanghai 200245, China; 5 Department of Chemistry, School of Life Sciences, University of Sussex, Brighton BN1 9QJ, UK; qiao.chen@sussex.ac.uk

**Keywords:** hexadecyl trimethyl ammonium bromide (CTAB), reduced graphene oxide (rGO), nanohybrid, NO_2_ gas-sensing properties, room temperature

## Abstract

A new NO_2_ nanohybrid of a gas sensor (CTAB-MoS_2_/rGO) was constructed for sensitive room-temperature detection of NO_2_ by 3D molybdenum disulfide (MoS_2_) and reduced graphene oxide (rGO), assisted with hexadecyl trimethyl ammonium bromide (CTAB). In comparison with MoS_2_ and MoS_2_/rGO, the BET and SEM characterization results depicted the three-dimensional structure of the CTAB-MoS_2_/rGO nanohybrid, which possessed a larger specific surface area to provide more active reaction sites to boost its gas-sensing performance. Observations of the gas-sensing properties indicated that the CTAB-MoS_2_/rGO sensor performed a high response of 45.5% for 17.5 ppm NO_2_, a remarkable selectivity of NO_2_, an ultra-low detection limit of 26.55 ppb and long-term stability for a 30-day measurement. In addition, the response obtained for the CTAB-MoS_2_/rGO sensor was about two to four times that obtained for the MoS_2_/rGO sensor and the MoS_2_ sensor toward 8 ppm NO_2_, which correlated with the heterojunction between MoS_2_ and rGO, and the improvement in surface area and conductivity correlated with the introduction of CTAB and rGO. The excellent performance of the CTAB-MoS_2_/rGO sensor further suggested the advantage of CTAB in assisting a reliable detection of trace NO_2_ and an alternative method for highly efficiently detecting NO_2_ in the environment.

## 1. Introduction

Although nitrogen dioxide (NO_2_) matters a lot in the industrial, farming and healthcare fields, it is also one kind of poisonous gas—harmful to environment and human health. In general, NO_2_ is generated from burning fossil fuels or automotive emissions, leading to a lot of serious diseases such as pulmonary, cardiovascular and cardiac diseases, even at low concentrations [1,2]. On this account, many analytical techniques have been applied for monitoring and detecting NO_2_, including electrochemical, optical, chemiresistive, spectroscopies methods and so on [3]. However, these techniques usually require time-consuming sample treatment procedures, expensive and bulky instruments that could not provide convenient and real-time online detection for the leaking, diffusing or transferring of NO_2_ gas [4,5]. Because of many superiorities, including low cost, easy operation and rapid, real-time detection and warning, portable semiconductor chemiresistive gas sensors can offer an alternative method for the effective monitoring and detecting of NO_2_ [6]. Nowadays, portable semiconductor chemiresistive gas sensors have been extensively applied for smart environmental monitoring and have shown a higher commercial application potential [7].

For the abundant vacancies and oxygen-containing functional groups on composite materials containing [8] graphene and derivatives, the sensors have been utilized for practical applications and have attracted considerable attention for detecting poisonous gas in environmental and industrial fields and monitoring exhaled air. Nowadays, rGO sensors have become one of the most common gas sensors. Wang et al. reported a novel NO_2_ sensor produced with the heterostructure of ZnO-SnO_2_ on rGO, which displayed a high response of 141.0% toward 5 ppm of NO_2_ at room temperature [9]. Liu et al. synthesized a NO_2_ sensor by ZnO-rGO hybrids, whose response towards 5 ppm NO_2_ operating at room temperature was 25.6%. The response time and recovery time towards 5 ppm were 165 s and 499 s, respectively [10]. Chan et al. prepared rGO-In_2_O_3_ hybrid nanostructure sensors, which presented a fine response of 22.3% toward 500 ppb NO_2_ at 150 °C [11]. Meanwhile, the two-dimensional layered nano-material of MoS_2_ also drew much attention on account of its prominent semiconductor properties and high surface-to-volume ratio [12]. Yang et al. described the main attributes of two-dimensional nanomaterials and demonstrated that an intense interaction existed between MoS_2_ and NO_2_ molecules, suggesting that MoS_2_ could be one special material for sensitively detecting NO_2_ [13,14]. To a certain extent, MoS_2_-based gas sensors exhibited better sensing properties than some carbon materials such as graphene and CNT [15]. However, MoS_2_-based gas sensors are still facing numerous challenges, such as short lifetime, tardy response or recovery rate, poor gas selectivity and complicated fabrication, which might limit their development or application [16,17,18,19,20].

Surfactants are known for their capability for controlling crystal morphology in the fabrication of inorganic nanomaterials [21]. CTAB is a common surfactant, and it is used extensively for synthesizing nanoparticles [22]. Zhang et al. synthesized a three-dimensional MoS_2_ nanoflower with a size of around 300 nm in the presence of CTAB, and the MoS_2_-based sensor displayed a response of 60% to 50 ppm NO_2_ at 100 °C, but it suffered a low response of almost 8% at room temperature [23]. Lin et al. reported MoS_2_/rGO composites synthesized with CTAB, which had a larger surface area than those synthesized using TritonX-100 or sodium dodecyl sulfonate [24]. Notably, the strategy of employing a surfactant for nanocomposite fabrication could improve gas adsorption on the material’s surface and promote gas-sensing performance.

In our work, a new CTAB-MoS_2_/rGO nanohybrid sensor was fabricated for room temperature, detecting NO_2_ based on hydrothermal and ultrasonic processes. Under ambient conditions, the gas-sensing performances of the CTAB-MoS_2_/rGO, MoS_2_ and MoS_2_/rGO sensors were systematically studied, showing that the response obtained for the CTAB-MoS_2_/rGO sensor was about four times that obtained for the MoS_2_ sensor and two times that obtained for the MoS_2_/rGO sensor to 8 ppm NO_2_. Under ambient conditions, the CTAB-MoS_2_/rGO sensor performed a high response of 45.5% for 17.5 ppm NO_2_, an ultralow detection limit of 26.55 ppb, excellent selectivity for NO_2_ and long-term stability for a 30-day persistent property test. The remarkable gas-sensing performance possibly owed to the formation of a heterojunction between the MoS_2_ and rGO, the increase in the number of active sites with the introduction of CTAB and the improvement of conductivity with rGO.

## 2. Materials and Methods

### 2.1. Materials and Chemicals

The reduced graphene oxide (rGO) dispersion was received from Chengdu Organic Chemicals Co. Ltd. (Chengdu, China) Thiourea (N_2_H_4_CS, 99%) and hexadecyl trimethyl ammonium bromide (CTAB, 99%) were obtained from Shanghai Macklin Biochemical Co. Ltd. (Shanghai, China). The sodium molybdate dihydrate (Na_2_MoO_4_·2H_2_O, 99%) was purchased from Shanghai Adamas-beta Co. Ltd. (Shanghai, China).

### 2.2. Sample Preparation

**Figure 1:**
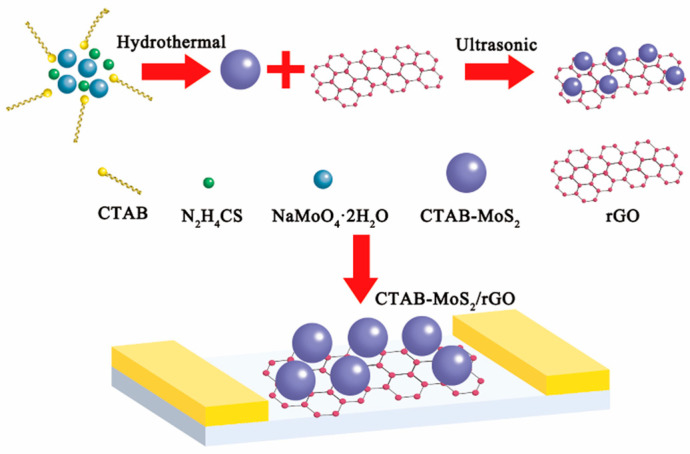
The preparation of CTAB-MoS_2_/rGO nanohybrid.

For the CTAB-MoS_2_ nanohybrid synthesis, in a typical run, 2 mmol NaMoO_4_·2H_2_O and 10 mmol thiourea were fully dispersed into 70 mL deionized water, then followed by sonication for 30 min. Subsequently, 210 mg CTAB was dissolved into the above solution through extra sonication for 30 min (the details about optimization and the XRD graph are in Figure S1). Next, the solution was poured into a 100 mL autoclave and kept at 210 °C for 24 h. The CTAB-MoS_2_ powder was obtained after centrifugation and washed with deionized water and anhydrous ethanol. The MoS_2_ was prepared with the same procedure without the CTAB. In the CTAB-MoS_2_/rGO fabrication process, 10 mg of the obtained CTAB-MoS_2_ powder was added into 1 mL ethanol, followed by sonication for 30 min in order to completely disperse. After completely dispersing, 0.1 mL of the rGO dispersion was dropped into the above solution under sonication for 1 h to synthesize the CTAB-MoS_2_/rGO hybrid. The MoS_2_/rGO composite was also synthesized with the same procedure. A diagram of the preparation of the composite is illustrated in Figure 1.

### 2.3. Characterization of Materials

The morphologies of the sensing materials were analyzed by a scanning electron microscope (SEM, SU8220, Hitachi, Tokyo, Japan) and a transmission electron microscope (TEM, JEM-2100F, JEOL). Crystallinity information was obtained by X-ray diffraction (XRD, D8 ADVANCE, Bruker, Cu kα, 380 eV). The composition information was characterized by X-ray photoelectron spectroscopy (XPS, ESCALAB 250, Al X-ray source, 0.5 eV). A confocal Raman microscope (in Via RENISHAW) was utilized to obtain Raman spectra.

### 2.4. Fabrication of CTAB-MoS_2_/rGO Sensor

The CTAB-MoS_2_/rGO sensor was fabricated on interdigital electrodes, which were composed of CTAB-MoS_2_/rGO sensing materials and interdigital electrodes. The interdigital electrodes were cleaned under sonication with acetone, deionized water and ethanol, respectively, before use. The composite suspension was obtained by adding 10 mg hybrid powder into 1 mL ethanol under sonication for 0.5 h. After that, 0.5 μL of the solution was dropped onto the cleaned interdigital electrodes by pipette. Then, the interdigital electrodes were kept in a 60 °C oven for 3 h to remove the extra ethanol.

### 2.5. Measurement of Gas-Sensing Properties

In order to observe the gas-sensing properties, homemade gas-sensing test equipment was constructed and demonstrated, which consisted of gas cylinders, a gas-sensing chamber, a plastic conduit and a resistance acquisition device (see Figure S2 for fabrication details). The gas cylinders were filled with dry air and dry NO_2,_ respectively. The gas-sensing chamber contained a 300 mL cylindrical cavity with several holes for gas injection and venting. The resistance of the sensors was obtained with a Keithley 2701 resistance acquisition device. The bubbling method was used to adjust the moisture of injected gas. Typically, dry air was passed through a cylindrical cavity with a certain volume of water to obtain a relative humidity (RH), and then mixed with dry NO_2_ [25,26]. Various concentrations of NO_2_ were gained by mixing the wet air with dry NO_2_, and all the gas flow ratios were regulated by mass flow controllers (MFC). The final flow rate of gas was fixed as 400 mL/min. The response value of the sensor was determined by (ΔR)/R_0_ × 100%, where ΔR = (R_0_ − R_g_), R_0_ was the resistance value in the air and R_g_ was the resistance value in NO_2_. For all the gas-sensing performance measurements, NO_2_ was introduced into the gas-sensing chamber for 10 min. The following sensing tests were implemented at room temperature and 58% RH, if without any special indication.

## 3. Results

### 3.1. Morphological and Structural Characterization

**Figure 2:**
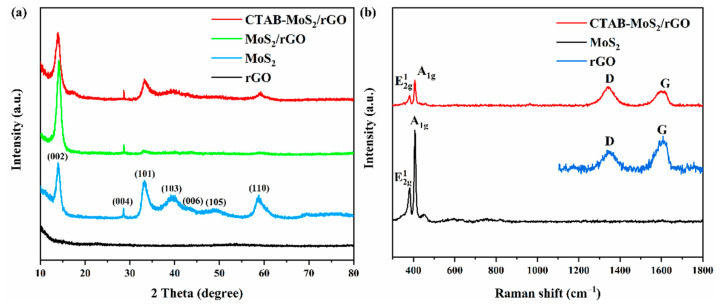
(**a**) XRD characterization of rGO, MoS_2_, MoS_2_/rGO and CTAB-MoS_2_/rGO; (**b**) Raman characterization of rGO, MoS_2_ and CTAB-MoS_2_/rGO.

Figure 2a suggests that the crystal planes of pure MoS_2_ were assigned to the (002), (004), (101), (103), (006), (105) and (110) of the hexagonal phase. It has been proven that 2H-MoS_2_ had better semiconductor properties and more thermal stability than 1T-MoS_2_ and 3R-MoS_2_ [27]. The (002) crystal plane indicated stacking a layered structure along the c-axis in bulk MoS_2_ [28]. When the rGO and CTAB were introduced to the hybrid, the main peaks of the (004), (101), (103), (006), (105) and (110) crystal planes became weaker or even disappeared, to restrain the aggregation and restacking of the layered structure of the MoS_2_ [29]. Meanwhile, no peak around 2 theta of 25 degrees appeared for the MoS_2_/rGO and CTAB-MoS_2_/rGO composites, which could suggest a decrease in the layer stacking of the rGO with the existence of MoS_2_.The XRD investigations demonstrated the prepared MoS_2_ was in accordance with JCPDS card 37–1492, and some peaks of the MoS_2_ became weaker or disappeared, which was beneficial for the promotion of sensing performance [30].

The structure of the CTAB-MoS_2_/rGO was carefully studied by Raman characterization. Figure 2b shows that two typical peaks at 380.9 cm^−1^ and 407.5 cm^−1^ were regarded as the in-plane vibration E^1^_2g_ and the out-of-plane vibration A^1^_g_ of the MoS_2_. The peak position of E^1^_2g_ and A^1^_g_ could be applied to calculate the layer numbers of the MoS_2_ [31]. The Raman shift difference was 26.6 cm^−1^, which could suggest that the MoS_2_ had a multilayer structure. Meanwhile, Figure 2b also depicts a D band (1348.39 cm^−1^) and a G band (1593.39 cm^−1^) of rGO, which indicated that the hybrid of MoS_2_/rGO was successfully formed by the introduction of rGO. In addition, the D band was induced by defects, and the G band was attributed to sp^2^ hybridized carbon atoms [32].

**Figure 3:**
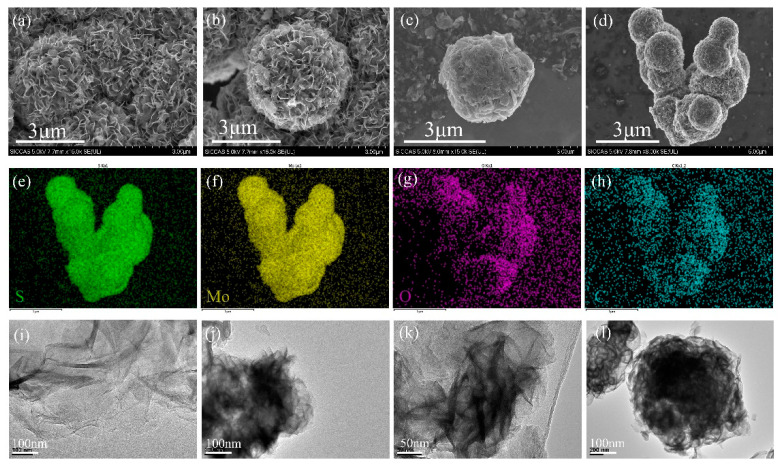
(**a**,**b**) SEM images of MoS_2_. (**c**,**d**) SEM images of MoS_2_/rGO and CTAB- MoS_2_/rGO. (**e**–**h**) The elemental mapping of CTAB-MoS_2_/rGO. TEM images of (**i**) rGO, (**j**) MoS_2_, (**k**) MoS_2_/rGO, (**l**) CTAB- MoS_2_/rGO.

The microstructures and morphologies of the sensing materials were further characterized through SEM. Figure 3a shows a three-dimensional flower-like structure of the MoS_2_ with multiple nanosheets. The thin nanosheets can be distinctly observed in Figure 3b. Figure 3c suggests that the rGO was closely attached to the MoS_2_ micro-flower for the formation of the MoS_2_/rGO hybrids, which was consistent with the investigation of Raman spectra. The image of the CTAB-MoS_2_/rGO is presented in Figure 3d, and the elemental mapping results are presented in Figure 3e–h. It was clear that the CTAB-MoS_2_/rGO material mainly contained C, O, Mo and S with homogeneous distribution. Furthermore, the TEM images in Figure 3i–l present the morphologies of the rGO, MoS_2_, MoS_2_/rGO and the CTAB-MoS_2_/rGO, respectively. The high-magnification TEM image further demonstrates that the molybdenum disulfide was multi-layered.

**Figure 4:**
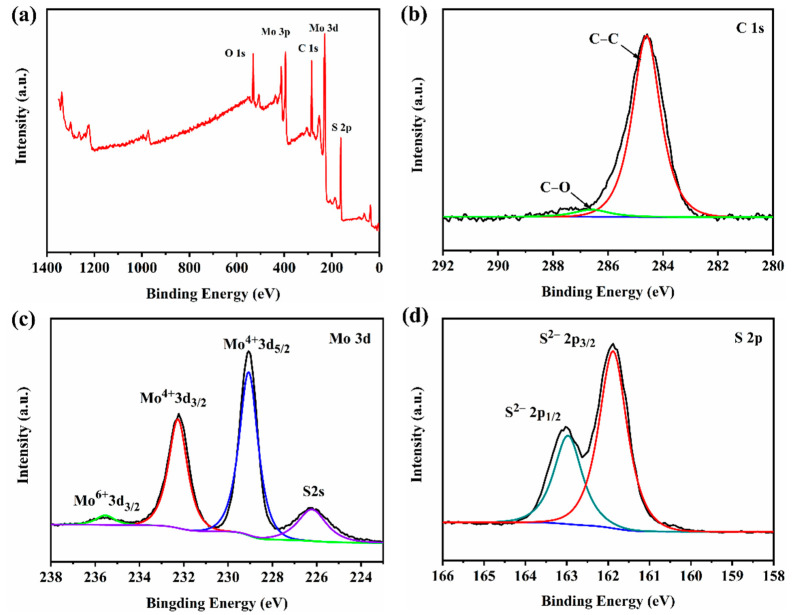
XPS spectra of CTAB-MoS_2_/rGO: (**a**) survey spectrum, (**b**) C 1s, (**c**) Mo 3d and (**d**) S 2p.

Figure 4a indicates the survey spectrum, suggesting the presence of C, Mo, S and O elements. Figure 4b demonstrates that the typical peaks at 284.6 and 286.6 eV could be assigned to C-C of the graphene skeleton and C-O bond [33]. Figure 4c suggests two valence states for Mo peaks. The peak at 232.2 eV could be assigned to Mo^4+^ 3d_3/2,_ and the peak at 229.1 eV was assigned to Mo^4+^ 3d_5/2_ [34]. The small peak at 235.4 eV could have resulted from Mo^6+^ 3d_3/2_, which was potentially ascribed to the low amount of MoO_3_ in the composite as reported in the previous literature [35]. The peak at 226.3 eV could be assigned to S 2 s. Moreover, Figure 4d suggests that the peaks of 163.0 eV and 161.9 eV could be assigned to S^2−^ 2p_1/2_ and S^2−^ 2p_3/2_ of the MoS_2_ [36].

### 3.2. Gas-Sensing Properties

**Figure 5:**
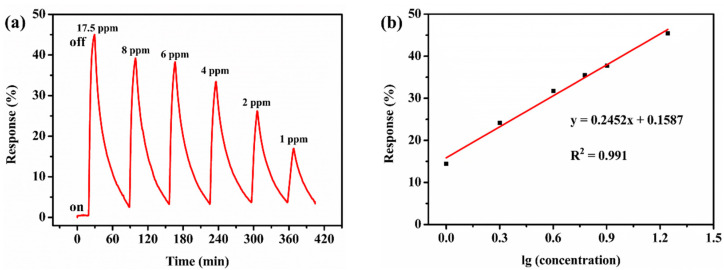
(**a**) Gas-sensing properties sensitivity of the CTAB-MoS_2_/rGO toward NO_2_ with concentration of 1–17.5 ppm at room temperature, RH of 58%. (**b**) The linear fitting curve between NO_2_ concentration and response for CTAB-MoS_2_/rGO.

The sequential dynamic reversible performance of the CTAB-MoS_2_/rGO sensor was investigated by exposing the CTAB-MoS_2_/rGO sensor toward various concentrations of NO_2_ of 1–17.5 ppm. The response values were 45.52%, 37.64%, 35.50%, 31.75%, 24.19% and 14.45% for 17.5 ppm, 8 ppm, 6 ppm, 4 ppm, 2 ppm and 1 ppm of NO_2_, respectively, which suggests that the CTAB-MoS_2_/rGO sensor could monitor NO_2_ in a wide concentration range sensitively. To further study the sensor’s properties, a linear fitting curve was plotted for the CTAB-MoS_2_/rGO sensor’s response values versus lg (NO_2_ concentration). Figure 5b suggests that the response values had an excellent linear relationship with lg (NO_2_ concentration), and its correlation coefficient (R^2^) was 0.991, which could demonstrate the reliable responses toward 1–17.5 ppm NO_2_. The detection limit in this work could be calculated by a formula [37]. By calculation, the LOD was 26.55 ppb, which was lower than the limit of 53 ppb claimed by the U.S. Environmental Protection Agency [38] (see Figure S3 for the calculation of the detection limit).

**Figure 6:**
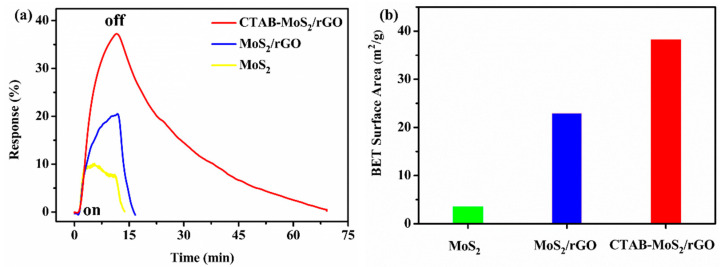
(**a**) The comparisons of the response and recovery properties of MoS_2_, MoS_2_/rGO and CTAB-MoS_2_/rGO sensors toward 8 ppm NO_2_ at room temperature and (**b**) the BET surface area of sensing materials.

Subsequently, the comparisons of the response and recovery properties were tested at room temperature and 58% RH for the CTAB-MoS_2_/rGO, MoS_2_/rGO and MoS_2_ sensors toward 8 ppm NO_2_. The MoS_2_ sensor performed a response of 9.7%. The MoS_2_/rGO sensor performed a response of 21.35%, which was two times that of the MoS_2_ sensor. The increased response demonstrated that the addition of rGO mattered a lot for improving the sensing properties, owing to the generation of a heterojunction between the MoS_2_ and rGO and the excellent performance of the rGO. More importantly, the CTAB-MoS_2_/rGO sensor displayed a response of 37.64% toward 8 ppm NO_2_, which was nearly two times the response for the MoS_2_/rGO sensor and four times the response for the pure MoS_2_ sensor. Moreover, the gradually increased specific surface area (Figure 6b) for the MoS_2_, MoS_2_/rGO and CTAB-MoS_2_/rGO might facilitate the distinct promotion of the gas-sensing properties.

**Figure 7:**
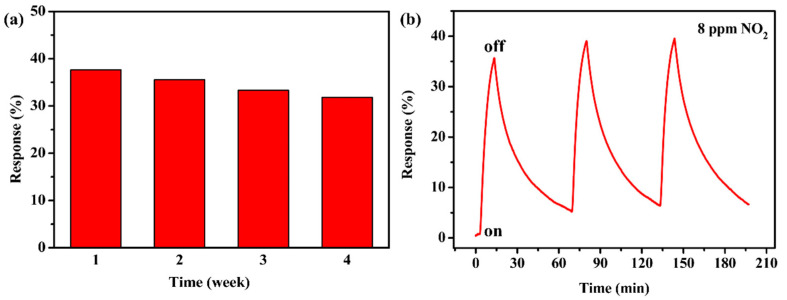
(**a**) Long-term stability of CTAB-MoS_2_/rGO sensor toward 8 ppm NO_2_ for four weeks and (**b**) repeatability of the CTAB-MoS_2_/rGO sensor.

Long-term stability was studied through continuously measuring the response values of the CTAB-MoS_2_/rGO sensor toward 8 ppm NO_2_ for four weeks (Figure 7a). The results showed that the response values decreased only 5.81% from 37.64% of the first week to 31.83% of the fourth week, and always maintained a high response to NO_2_ during the four weeks. The results indicated the gas sensor was capable of detecting NO_2_ with a remarkable repeatability and superior long-term stability.

The repeatability was tested by exposing the CTAB-MoS_2_/rGO sensor to 8 ppm NO_2_ for three response–recovery cycles at room temperature and 58% RH (Figure 7b). It was noticeable that the response values maintained practically the same values after three cycles without an obvious response drop, which could confirm splendid repeatability for the CTAB-MoS_2_/rGO sensor.

**Figure 8:**
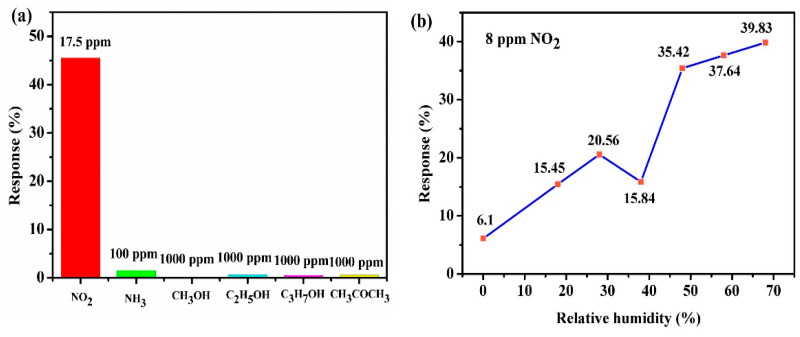
(**a**) Selectivity of CTAB-MoS_2_/rGO sensor toward various gases, and (**b**) humidity influence on response for CTAB-MoS_2_/rGO sensor toward 8 ppm NO_2_.

Since selectivity is a crucial property for gas sensors in real environments, the selectivity of the CTAB-MoS_2_/rGO sensor was measured by exposing it to 17.5 ppm of NO_2_, 100 ppm of NH_3_, 1000 ppm of methanol, ethanol, isopropanol and acetone. Figure 8a shows that the response value toward 17.5 ppm NO_2_ was far higher than the higher concentration of all of the interfering gas species, suggesting an outstanding selectivity of the CTAB-MoS_2_/rGO sensor toward NO_2_. The extremely low response to high concentrations of methanol, ethanol, isopropanol and acetone may have resulted from the high activation energy barrier of the reaction between the sensing material surface and VOCs [39].

The influence of RH on gas-sensing properties was further explored by exposing the CTAB-MoS_2_/rGO sensor toward 8 ppm NO_2_ at different moisture levels (0–68% RH), and the result is shown in Figure 8b. Here, RH in the gas-sensing chamber was controlled by letting dry air pass through a cylindrical cavity of water and mixing it with dry NO_2_. Before injecting NO_2_ into the gas-sensing chamber, the CTAB-MoS_2_/rGO sensor was exposed to wet air with a desired relative humidity. After the resistance was relatively stable, 8 ppm NO_2_ was introduced to the gas chamber. The sensor response (6.1%) was very low when exposed to 8 ppm of dry NO_2_. As the relative humidity rose from 0 to 28%, the response rose from 6.1% to 20.56% accordingly. While increasing the moisture level to 38% RH, the response decreased to 15.84%, which could be due to the reduction in the number of active reaction sites. Since the gas adsorption sites on the MoS_2_/rGO were occupied by abundant water, the number of active reaction sites and the sensitivity decreased. The response increased dramatically as the relative humidity continued to increase to a higher moisture level (38–68% RH). It is possible that the electrons of CTAB-MoS_2_/rGO were captured by NO_2_ due to its higher electrophilic property to form NO_2_^−^, and NO_2_^−^ simultaneously reacted with the adsorbed oxygen ion to form NO_3_^−^. NO_3_^−^ was dominant even though NO_2_^−^ and NO_3_^−^ could exist at the same time when the moisture level increased to a high relative humidity. The binding energy of NO_3_^−^ was higher than NO_2_^−^, which could result in the enhancement of the response value at a higher moisture level [40]. However, when the relative humidity was at 48–68%, the response values were almost similar, which was possibly caused by the saturation of the impact of the relative humidity on response. The results indicated that the sensors showed excellent gas-sensing properties in high-RH ambient, qualifying them for detecting NO_2_ gas in a high-RH environment. In addition, the CTAB-MoS_2_/rGO sensor showed excellent sensitivity and a low detection limit at room temperature compared to the previous TMDs-based sensors and rGO-based sensors (Table 1).

## 4. Discussion

**Figure 9:**
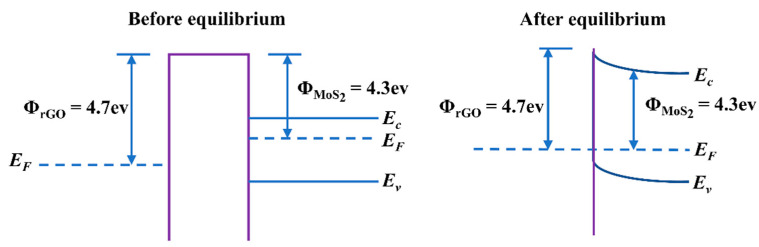
The diagram of energy band at CTAB-MoS_2_/rGO interfaces.

According to previous reports [49], the active species NO_3_^-^ originated from NO_2_ may play a significant role in causing the resistance changes of sensing materials. Based on the theory and the experiments [50], a possible mechanism was proposed and illustrated for the CTAB-MoS_2_/rGO sensor (Figure 9). When the CTAB-MoS_2_/rGO sensor was exposed to the air, the oxygen molecules in the air would adsorb electrons in the gas-sensitive material to form chemically adsorption oxygen O_2_^−^. When the CTAB-MoS_2_/rGO hybrid contacted NO_2_ gas, the electrons would transfer from the hybrid to NO_2_ to form NO_2_^−^, resulting in the rise of hole concentration and the decline of resistance. Meanwhile, NO_2_ with strong electron absorption ability could also seize electrons from the material and react with the previous O_2_^−^ to generate NO_3_^−^. While the sensor was exposed to air again, NO_3_^−^ would desorb from the sensing material surface, and the electron would return to the hybrid, thus the resistance would recover to the initial state. Moreover, the formation of the heterojunction between the MoS_2_ and rGO shows that electrons are transported from the conduction band of MoS_2_ to that of rGO [51]. The increase in the specific surface area with CTAB and the improvement of conductivity with rGO might result in the enhancement of the gas-sensing properties of the CTAB-MoS_2_/rGO sensor. The whole reaction is as follows:

O_2_(gds) → O_2_(ads) (1)

O_2_(ads) + e^−^ (from CTAB-MoS_2_/rGO) → O_2_^−^(ads) (2)

NO_2_(gas) + e^−^ (from CTAB-MoS_2_/rGO) → NO_2_^−^(ads) (3)

2NO_2_(gas) + O_2_^−^(ads) + e^−^ (from CTAB-MoS_2_/rGO) → 2NO_3_^−^(ads) (4)

2NO_3_^−^(ads) → 2NO_2_(gas) + O_2_(ads) + e^−^ (to CTAB-MoS_2_/rGO) (5)

3NO_2_(gas) + H_2_O → NO(gas) + 2H^+^ + 2NO_3_^−^(ads) (6)

## 5. Conclusions

In the present work, an efficient CTAB-MoS_2_/rGO gas sensor was successfully prepared for room-temperature detection of NO_2_ with the assistance of surfactant CTAB. The sensing properties of the MoS_2_, MoS_2_/rGO and CTAB-MoS_2_/rGO sensors were systematically investigated by exposure to NO_2_. The introduction of rGO and surfactant CTAB could significantly promote sensitivity to NO_2_, and the capability of recovery was slightly decreased due to the effect of rGO. The investigations demonstrated that the CTAB-MoS_2_/rGO sensor manifested an excellent response, remarkable repeatability, long-term stability and selectivity toward NO_2_. The great sensing performance could result from the generation of a heterojunction, the positive effect of CTAB and the good conductivity of rGO. The investigations also suggested that the surfactant was a great prospect for fabricating high properties of room-temperature gas sensors. Further research in this area is being implemented in our laboratory.

## Figures and Tables

**Table 1:** Comparison of previously reported TMDs-based sensors, rGO-based sensors and CTAB-MoS_2_/rGO sensor.

Materials	Temperature (°C)	Concentration (ppm)	Response	LOD (ppb)	References
MoS_2_/CNT	RT	25	5% ^a^	25	[41]
ZnO-rGO	RT	5	25.6% ^a^		[9]
NbS_2_	RT	5	18% ^a^	241.02	[42]
MoS_2_/SnO_2_	RT	5	5% ^a^	500	[43]
MoS_2_/rGO	RT	5	14.28% ^a^	50	[32]
rGO/Co_3_O_4_	RT	5	26.8% ^a^	50	[44]
SnS_2_/rGO	RT	8	49.8% ^b^	8.7	[45]
rGO/ZnO	RT	15	45% ^a^		[46]
MoS_2_/ZnO	RT	5	3050% ^c^	50	[47]
In_2_O_3_/MoS_2_	RT	10	50 ^d^	8.8	[48]
CTAB-MoS_2_/rGO	RT	8	37.64% ^a^	26.55	Our work

^a^ (R_0_ − R_g_)/R_0_; ^b^ ΔG/G_0_; ^c^ (I_g_ − I_0_)/I_0_; ^d^ R_g_/R_0_. RT = room temperature.

## Data Availability

The data presented in this study are available on request from the corresponding author.

## Supplementary Materials

The following are available online at https://www.mdpi.com/article/10.3390/nano12081300/s1, Figure S1. XRD of different concentrations of CTAB-MoS2, Figure S2. SEM images of CTAB-MoS_2_; (a) 0 mg/mL (b) 1.5 mg/mL (c) 3 mg/mL (d) 6 mg/mL, Figure S3. Schematic image of the gas sensor measurement system, Table S1. A fifth order polynomial fitting result.
