# Supplementary material for: CTAB Enhanced Room-Temperature Detection of NO2 Based on MoS2-Reduced Graphene Oxide Nanohybrid
Source: Nanomaterials (Basel). 2022 Apr 11;12(8):1300. doi: 10.3390/nano12081300 (PMC9032584; doi:10.3390/nano12081300)
Supplement: Supplementary file 1 [file nanomaterials-12-01300-s001.zip › nanomaterials-1646881-supplementary.pdf]

nanomaterials

MDPI

Supplementary Materials

CTAB Enhanced Room-Temperature Detection of NO_2 Based on MoS_2-Reduced Graphene Oxide Nanohybrid

Wenbo Li [1,2,3], Hao Li [1,2,3], Rong Qian [1,3,*], Shangjun Zhuo [1,3], Pengfei Ju [4,*] and Qiao Chen [5]

[1] National Center for Inorganic Mass Spectrometry in Shanghai, Shanghai Institute of Ceramics, Chinese Academy of Sciences, Shanghai 200050, China; hymnek@hotmail.com (W.L.), lh960714211@163.com (H.L.), sjzhuo@mail.sic.ac.cn (S.Z.)
[2] School of Material Science and Engineering, University of Shanghai for Science and Technology, Shanghai 200093, China
[3] Center of Materials Science and Optoelectronics Engineering, University of Chinese Academy of Sciences, Beijing 100049, China
[4] Shanghai Aerospace Equipment Manufacturer, Shanghai 200245, China
[5] Department of Chemistry, School of Life Sciences, University of Sussex, Brighton BN1 9QJ, UK; Qiao.Chen@sussex.ac.uk
* Correspondence: qianrong@mail.sic.ac.cn (R.Q.); jupengfei10@163.com (P.J.)

S1. The details about optimisation and XRD graph.

In this study, we studied the effects of different concentrations of CTAB on molybdenum disulfide. XRD characterization of CTAB-MoS_2 with different concentrations (0, 1.5, 3, 6 mg/mL) of CTAB were shown as following. The following Figure S1 suggested that the main crystal faces of CTAB-MoS_2 in different concentrations could match the standard card of MoS_2, which could indicate that the prepared material was MoS_2.

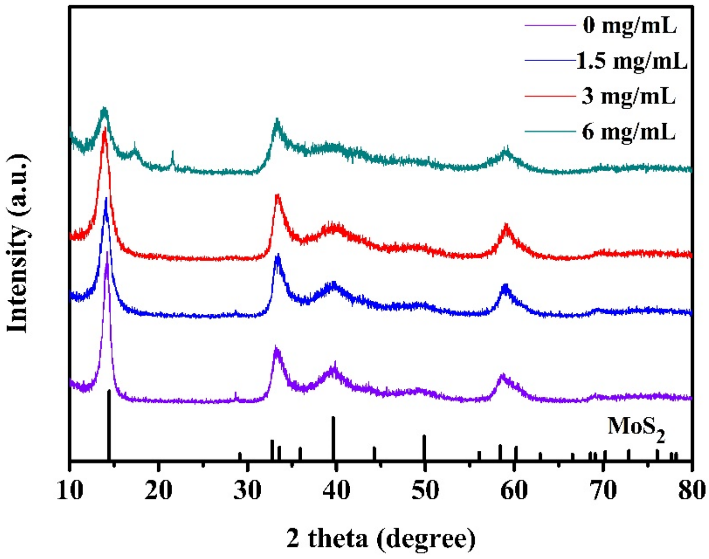

Figure S1. XRD of different concentrations of CTAB-MoS_2.

In order to achieve a better gas sensitivity of the NO_2 sensor with CTAB, four nanohybrid materials with the concentration of CTAB of 0 mg/mL (a), 1.5 mg/mL (b), 3 mg/mL (c), and 6 mg/mL (d) were farbricated and characterized by SEM. In Figure S2a, the size of MoS_2 without CTAB was about 6.5 µm, and the MoS_2 microspheres were agglomerated together. It is possible that large size might reduce the specific surface area, and severe agglomeration was not beneficial to the adsorption of the gas on the surface of the material to probably reduce the gas sensitivity. In Figure S2b, MoS_2 agglomeration seemed to be more severe when the concentration of CTAB was 1.5 mg/mL, and there was no clear spherical MoS_2. In Figure S2c, when the concentration of CTAB was 3 mg/mL, it was clear

that MoS_2 presented a spherical structure with visible nanosheets on the surface, and the size of MoS_2 microsphere was about 3 μm. In Figure S2d, when the concentration of CTAB was increased to 6 mg/mL, some unsphered flaky MoS_2 emerged without good dispersion. Based on SEM analysis, the CTAB concentration of 3 mg/mL was selected as the preferred concentration for the following experiments.

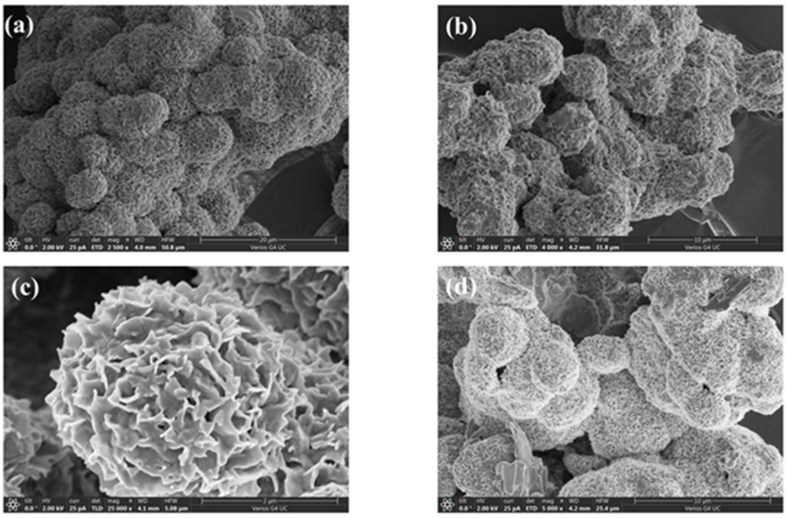

Figure S2. SEM images of CTAB-MoS_2; (**a**) 0 mg/mL (**b**) 1.5 mg/mL (**c**) 3 mg/mL (**d**) 6 mg/mL.

S2. The diagram of homemade gas-sensing investigation system.

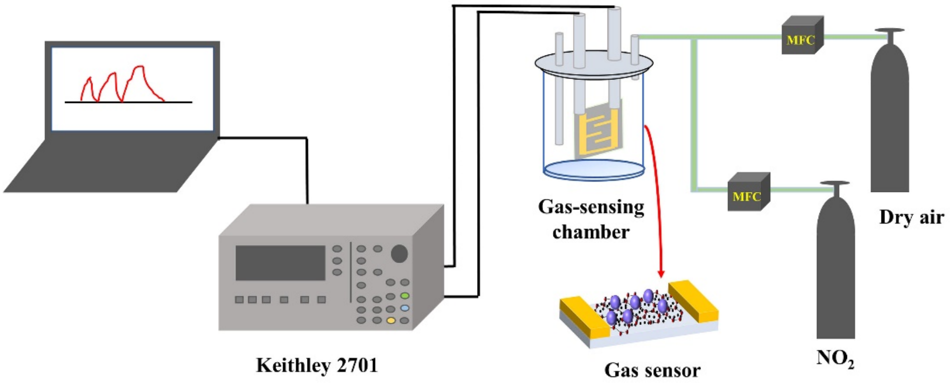

Figure S3. Schematic image of the gas sensor measurement system.

S3. The calculation of the detection limit

More details about the calculation of the detection limit of the CTAB-MoS_2/rGO NO_2 sensor is presented as following:

1. The noise of the sensor is calculated by root mean square deviation (rmsd) using the change of relative conductivity at baseline. 10 data points were collected at baseline before NO_2 exposure. After plotting the data, a fifth order polynomial fitting is carried out in the range of data points.

In eq 1, y_i is the measured data point and y is the corresponding value calculated from the curve-fitting equation.

$$V_{X^2} = \sum(y_i - y)^2 \quad (1)$$

Copyright: © 2022 by the authors. Licensee MDPI, Basel, Switzerland. This article is an open access article distributed under the terms and conditions of the Creative Commons Attribution (CC BY) license (https://creativecommons.org/licenses/by/4.0/).

Table S1. A fifth order polynomial fitting result.

Time (s)	yi	y	yi−y	(yi−y)^2
250	4.24×10^{-4}	-3.29×10^{-4}	7.53×10^{-4}	5.67×10^{-7}
260	1.49×10^{-3}	4.49×10^{-3}	-3.00×10^{-3}	9.00×10^{-6}
270	8.31×10^{-3}	5.04×10^{-3}	3.27×10^{-3}	1.07×10^{-5}
280	5.34×10^{-3}	3.96×10^{-3}	1.38×10^{-3}	1.92×10^{-6}
290	-1.41×10^{-3}	2.84×10^{-3}	-4.24×10^{-3}	1.79×10^{-5}
300	2.91×10^{-3}	2.40×10^{-3}	5.13×10^{-4}	2.63×10^{-7}
310	5.03×10^{-3}	2.65×10^{-3}	2.38×10^{-3}	5.66×10^{-6}
320	2.36×10^{-3}	3.07×10^{-3}	-7.06×10^{-4}	4.98×10^{-7}
330	2.09×10^{-3}	2.76×10^{-3}	-6.62×10^{-4}	4.38×10^{-7}
340	9.56×10^{-4}	6.58×10^{-4}	2.96×10^{-4}	8.78×10^{-8}

2. The blank noise is calculated as

$$RMS_{noise} = \sqrt{\frac{V_{X^2}}{N}} \tag{2}$$

In eq 2, RMS_{noise} is the blank noise, and N is the number of data points collected at baseline. The CTAB-MoS_2/rGO sensor blank noise is calculated as 4.71×10^{-6}.

3. Based on the above data and according to the definition of detection limit (DL) calculation equation (Equation 3), the theoretical detection limit (DL) of CTAB-MoS_2/rGO sensor is calculated to be 26.55 ppb.

$$LOD = 3\frac{RMS_{noise}}{Slope} \tag{3}$$
